# Bioconversion of methane to lactate by an obligate methanotrophic bacterium

**DOI:** 10.1038/srep21585

**Published:** 2016-02-23

**Authors:** Calvin A. Henard, Holly Smith, Nancy Dowe, Marina G. Kalyuzhnaya, Philip T. Pienkos, Michael T. Guarnieri

**Affiliations:** 1 National Bioenergy Center, National Renewable Energy Laboratory, Golden, CO 80401 USA; 2 Department of Biology, San Diego State University, San Diego, CA 92182 USA

## Abstract

Methane is the second most abundant greenhouse gas (GHG), with nearly 60% of emissions derived from anthropogenic sources. Microbial conversion of methane to fuels and value-added chemicals offers a means to reduce GHG emissions, while also valorizing this otherwise squandered high-volume, high-energy gas. However, to date, advances in methane biocatalysis have been constrained by the low-productivity and limited genetic tractability of natural methane-consuming microbes. Here, leveraging recent identification of a novel, tractable methanotrophic bacterium, *Methylomicrobium buryatense*, we demonstrate microbial biocatalysis of methane to lactate, an industrial platform chemical. Heterologous overexpression of a *Lactobacillus helveticus* L-lactate dehydrogenase in *M. buryatense* resulted in an initial titer of 0.06 g lactate/L from methane. Cultivation in a 5 L continuously stirred tank bioreactor enabled production of 0.8 g lactate/L, representing a 13-fold improvement compared to the initial titer. The yields (0.05 g lactate/g methane) and productivity (0.008 g lactate/L/h) indicate the need and opportunity for future strain improvement. Additionally, real-time analysis of methane utilization implicated gas-to-liquid transfer and/or microbial methane consumption as process limitations. This work opens the door to develop an array of methanotrophic bacterial strain-engineering strategies currently employed for biocatalytic sugar upgrading to “green” chemicals and fuels.

Methane (CH_4_), the primary component of natural gas and anaerobic digestion-derived biogas, offers a promising, high-volume petroleum replacement for fuel and chemical bioprocesses. Recent advances in gas-recovery technologies have facilitated access to previously inaccessible natural gas reserves, while biogas generated from anaerobic digestion of waste streams offers a versatile, renewable CH_4_ source. However, the gaseous state of CH_4_ makes for a lack of compatibility with current transportation and industrial manufacturing infrastructure, limiting its utilization as a transportation fuel and intermediate in biochemical processes. Importantly, CH_4_ is also the second most abundant greenhouse gas (GHG), with nearly 60% of emissions derived from anthropogenic sources^1^. Microbial conversion of CH_4_ to value-added chemicals using natural CH_4_-consuming bacteria offers valorization potential^2^,^3^,^4^, while reducing GHG emissions.

Obligate methanotrophic bacteria (methanotrophs) are a unique group of microorganisms capable of utilizing CH_4_ or methanol (CH_3_OH) as their sole carbon and energy source. These bacteria use the enzyme methane monooxygenase (MMO) to convert CH_4_ to CH_3_OH, which is further oxidized to formaldehyde (CH_2_O), formate (CHOOH) and CO_2_. Depending on the metabolic arrangement, CH_4_-derived carbon is assimilated at the level of CH_2_O (via the Ribulose-monophosphate cycle), methylene tetrahydrofolate and CO_2_ (Serine cycle), or CO_2_ (Calvin cycle)^5^,^6^. In the past, methanotrophs have been exploited for the conversion of CH_4_ to an array of products^7^, including bioprotein^8^,^9^, polyhydroxybutyrate^10^, carotenoids^11^,^12^,^13^, vitamins^14^, and CH_3_OH^15^,^16^. However, advances in CH_4_ biocatalysis and methanotroph strain engineering have largely been limited by the low-productivity of methanotroph cultures and lack of genetic tools for use in these organisms^3^,^7^,^17^.

Recently, an active Embden–Meyerhof–Parnas (EMP) pathway was identified in novel gammaproteobacterial methanotrophs that are resistant to the toxic components of natural gas and biogas^18^,^19^,^20^,^21^, and a set of genetic tools, including expression vectors, have been developed for the halotolerant, alkaliphilic methanotrophic bacteria *Methylomicrobium buryatense*^21^,^22^. Given the conserved nature of their downstream metabolic machinery, conventional industrial strain-engineering routes from sugars to biochemical intermediates and products can potentially be paralleled in these methanotrophs. Here, we report microbial biocatalysis of methane to an industrial platform chemical, lactate, a precursor to the biodegradable polylactide (PLA) polymer used in bioplastics. We demonstrate effective genetic engineering strategies in a methanotrophic bacterium, enabling production of lactate from both CH_4_ and CH_3_OH as sole carbon sources. The presented route circumvents competition with food substrates, such as corn, utilized in conventional sugar-based lactate production, and offers a potentially transformational path to concurrent mitigation of GHG emissions and biological CH_4_ upgrading.

## Results

### *M. buryatense* tolerance to lactate

**Figure 1:**
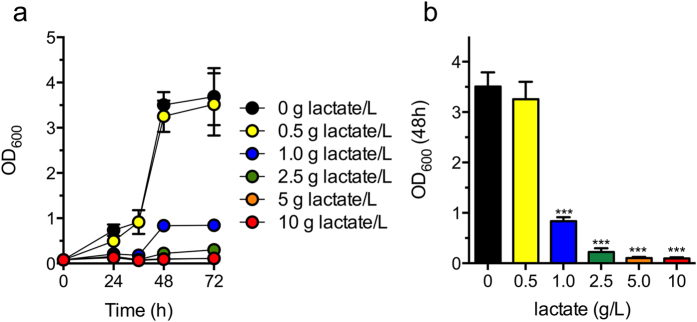
Lactic acid minimum inhibitory concentration against *M. buryatense*. Growth of *M. buryatense* in NMS2 medium supplemented with 1% CH_3_OH (v/v) and increasing concentrations of sodium lactate. Cultures were inoculated at OD_600_ = 0.1. The data represent the mean OD_600_ ± SEM of biological triplicates. ***p < 0.001.

In order to assess potential end product inhibition on bacterial growth, we first examined *M. buryatense* tolerance to increasing concentrations of sodium lactate in NMS2 medium^22^ containing CH_3_OH as the sole carbon source. Growth inhibition was observed at concentrations above 0.5 g lactate/L (Fig. 1), though this was not due to changes in the pH of the alkaline medium. These data suggest production of lactate from CH_4_ or CH_3_OH is feasible in *M. buryatense*, but also indicate that it may be difficult to achieve high lactate titers without addressing lactate toxicity.

### Construction of an inducible broad host-range vector for fine-tuned gene expression in *Methylomicrobium*

**Figure 2:**
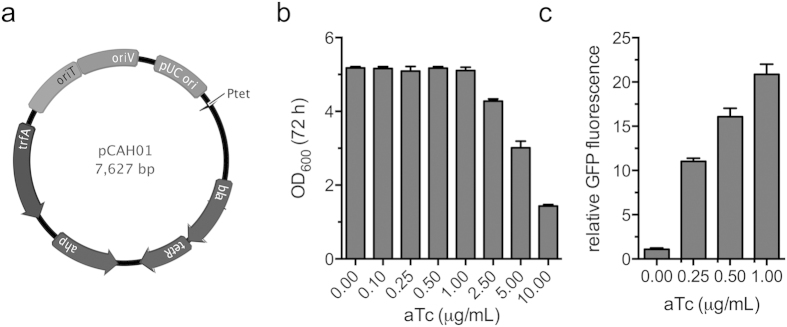
Characterization of the inducible, broad host range vector pCAH01. (**a**) pCAH01 was constructed by fusing the IncP-based origin of pAWP78 with the tetracycline promoter/operator (*P*_*tet*_) of pASK75 using Gibson assembly. *ahp* (kanamycin resistance), *bla* (ampicillin resistance), *tetR* (transcriptionally-fused tetracycline repressor), *oriV/oriT* (IncP-based origin of replication/transfer), *trfA* (*oriV* replication initiation protein). (**b**) Antimicrobial activity of the tet^p/o^ inducer, anhydrotetracycline, against wild-type *M. buryatense*. (**c**) tet^p/o^-dependent induction of GFP in *M. buryatense* grown in 1% CH_3_OH (v/v) NMS2 medium supplemented with increasing concentrations of anhydrotetracycline. Relative fluorescence was calculated by comparing fluorescence in pCAH01::emGFP samples to fluorescence in uninduced controls. The data in (**b**,**c**) represent the mean ± SEM from two independent experiments (n = 4).

Genetic tools for methanotrophic bacteria are currently limited, making heterologous gene expression and knockout difficult in these organisms. Although constitutive promoters functional in *M. buryatense* have recently been characterized^22^, an inducible promoter has yet to be identified for use in this genus. In order to facilitate regulated, heterologous gene expression in the *M. buryatense*, we constructed an inducible, broad-host range vector, pCAH01, (Fig. 2a) by fusing the tetracycline promoter/operator (tet^p/o^) from pASK75 with the IncP-based pAWP78 vector that can be replicated by *Methylomicrobium* spp.^22^,^23^. Anhydrotetracycline (aTc) is a tetracycline derivative commonly used as an inducer of the tet^p/o^ in bacteria^24^, which can exhibit antimicrobial activity at higher concentrations. We observed no effect on *M. buryatense* growth in the presence of 0.1–1.0 μg aTc/mL, whereas 2.5–10 μg aTc/mL inhibited bacterial growth (Fig. 2b). Experiments using GFP fluorescence as a readout of promoter activity indicated tightly controlled tet^p/o^-mediated gene expression in *M. buryatense* after induction with sub-lethal concentrations of the aTc inducer (Fig. 2c). Importantly, the tet^p/o^ did not show any “leaky” gene expression in the absence of inducer, making it a promising tool for conditional gene expression/knock-out studies in methanotrophic bacteria that replicate vectors containing the *oriV* origin of replication.

### Engineering of *M. buryatense* for lactate production

**Figure 3:**
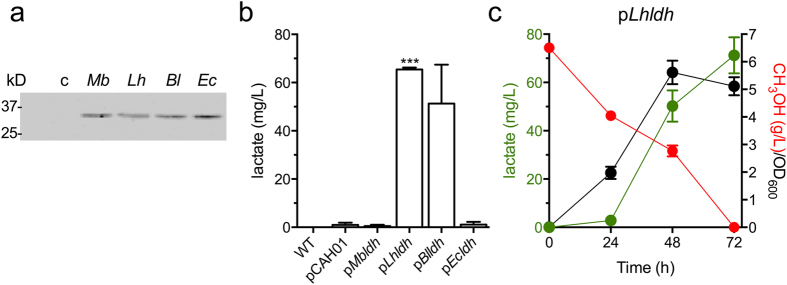
LDH expression and lactate accumulation in engineered strains of *M. buryatense*. Engineered *M. buryatense* harboring the pCAH01 empty vector (**c**) or ectopically expressing the native LDH (*Mb*), or heterologous, codon-optimized *L. helveticus* LDH (*Lh*), *B. longum* LDH (*Bl*), or *E. coli* LDHA (*Ec*) with C-terminal Flag tags were grown in shake flasks with 1% CH_3_OH (v/v). (**a**) Anti-Flag immunoblot confirmation of LDH expression 24 h post-induction. (**b**) Lactate titers in culture supernatants of induced strains 72 h post-inoculation/induction from aerobic shake flask cultures as measured by HPLC. (**c**) Growth (black), CH_3_OH consumption (red) and lactate accumulation (green) in shake flasks cultures of the *Lactobacillus helveticus* LDH-overexpressing methanotroph (p*Lhldh*). The data in (**b**,**c**) represent the mean ± SEM from at least two independent experiments (n = 2–4). ***p < 0.001.

We next employed pCAH01 to demonstrate methanotrophic biocatalysis targeting production of lactate. The biosynthetic conversion of the glycolytic intermediate pyruvate to lactate is catalyzed by an NADH-dependent lactate dehydrogenase (LDH) enzyme. Given that gammaproteobacterial methanotrophs have high flux of C1 substrates through pyruvate^19^, we hypothesized that heterologous LDH expression would facilitate CH_4_ biocatalysis to lactate in *M. buryatense*. Heterologous, codon-optimized LDH genes from *Escherichia coli*, *Bifidobacterium longum*, and *Lactobacillus helveticus*, whose corresponding LDHs have been used for the production of optically pure lactate^25^,^26^,^27^, as well as the native *M. buryatense ldh* gene, were placed under control of the tet^p/o^ with a 3′ Flag-tag to track protein expression (Table S1). Western blots using an anti-Flag antibody showed detectable LDH expression in all engineered strains post-induction (Fig. 3a).

We evaluated lactate production by the LDH-overexpressing strains under aerobic growth conditions since CH_4_ biocatalysis is oxygen dependent. Engineered strains expressing the *B. longum* or *L. helveticus* LDH accumulated lactate in the medium of CH_3_OH-grown aerobic shake flask cultures, whereas lactate production was not observed in strains overexpressing the native *M. buryatense* LDH, *E.coli* LDH, an empty vector control strain (Fig. 3b), or uninduced controls (data not shown). The *L. helveticus* LDH-expressing methanotroph (p*Lhldh*) produced significant levels of lactate (65 ± 1 mg/L after 72 h, *p* < 0.001 compared to wild-type) during the initial screen; therefore, it was selected for further analysis. The p*Lhldh* strain produced negligible lactate (~3 mg/L) during the first 24 h of growth, although it consumed 2.5 g/L CH_3_OH (Fig. 3c) and possessed significant LDH activity compared to wild-type *M. buryatense* during this period of growth (Table 1). In contrast, this strain accumulated 71 ± 15 mg lactate/L with a 0.018 ± 0.005 g lactate/g CH_3_OH yield between 24–72 h. Similar lactate titers were observed in CH_4_-grown shake-flask cultures (62 ± 35 mg/L, Figure S1).

### Bioconversion of methane to lactate by an engineered methanotrophic biocatalyst

**Figure 4:**
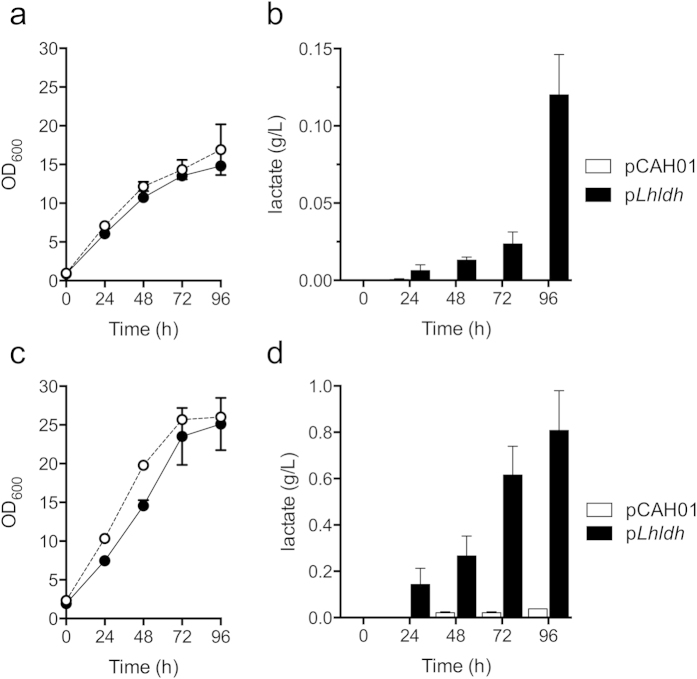
Growth and lactate production in continuously stirred tank methane bioreactors. Growth and lactate accumulation were monitored by engineered *M. buryatense* harboring an empty vector (pCAH01, open symbols/bars), or ectopically expressing heterologous *Lactobacillus helveticus* LDH (p*LhLDH*, closed symbols/bars) grown in a 0.5 L bioreactor (0.3 L culture volume, (**a**,**b**) or a 5.0 L bioreactor (3.0 L culture volume, (**c**,**d**) with continuous methane feed. The data represent the mean ± SEM from at least two independent experiments (n = 2–4).

**Figure 5:**
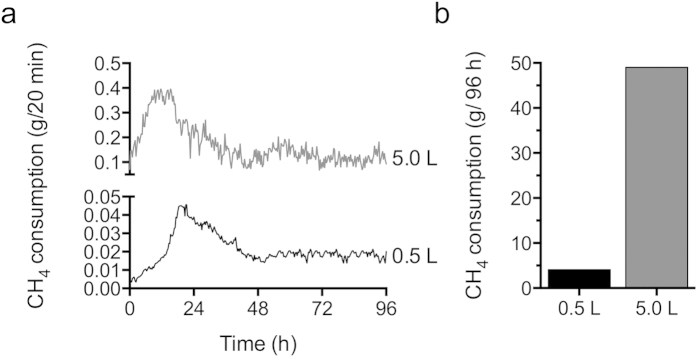
Methane consumption by engineered *M. buryatense*. (**a**) Real-time or (**b**) total methane consumption after 96 h by an engineered *M. buryatense* strain expressing heterologous *Lactobacillus helveticus* LDH (p*LhLDH*) in a 0.5 L or 5.0 L bioreactor. Data in (**a**,**b**) are representative data from at least two experiments.

We next evaluated growth and lactate production by *pLhldh* in both 0.5 L (Fig. 4a,b) and 5 L (Fig. 4c,d) continuously stirred tank bioreactors with constant CH_4_ feed (20% v/v CH_4_ in air) and increased nitrate, phosphate, and trace elements in the medium to support enhanced cell growth. As shown in Fig. 4a, a control strain carrying an empty pCAH01 vector and the *pLhldh* strain grew to high cell densities (OD_600_ ~17 and 15 after 96 h, respectively) in modified NMS2 medium at 0.5 L scale. The *pLhldh* strain produced 0.12 g lactate/L after 96 h of growth while no detectable lactate was produced by the induced control strain (Fig. 4b).

High cell-density cultures were also obtained in the 5 L bioreactor (OD_600_ ~25 for both control and p*Lhldh* strains, Fig. 4c). Here, the induced control strain accumulated low lactate titers (0.038 ± 0.0007 g lactate/L) in the larger bioreactor (Fig. 4d), presumably from native LDH activity. Lactate produced by the *pLhldh* strain reached titers of 0.808 ± 0.343 g lactate/L after 96 h, representing a 21-fold increase in lactate production over the control strain (Fig. 4d). Notably, these lactate titers coincide with the maximum lactate concentration tolerated by this organism (Fig. 1). To determine the lactate yield from CH_4_, we measured real-time CH_4_ consumption in the 0.5 L and 5 L bioreactor (Fig. 5a). The *pLhldh* strain consumed 4.06 g CH_4_ and 49.03 g CH_4_ after 96 h of growth in the 0.5 L and 5 L bioreactor, respectively (Fig. 5b), which was similar to the control strain (data not shown). Under our experimental conditions, a maximum 0.05 g lactate/g CH_4_ yield and 0.008 g/L/h volumetric productivity was observed from the *pLhldh* strain in a 5 L bioreactor (Table 2). Of note, 50–60% of consumed CH_4_ was utilized for biomass synthesis while additional carbon was lost to formate and acetate, which were detected in the medium of pCAH01 and p*Lhldh* strains under these growth conditions (Figure S2).

### Effect of lactate production on biosynthesis of potential methanotroph lipid-fuel precursors

**Figure 6:**
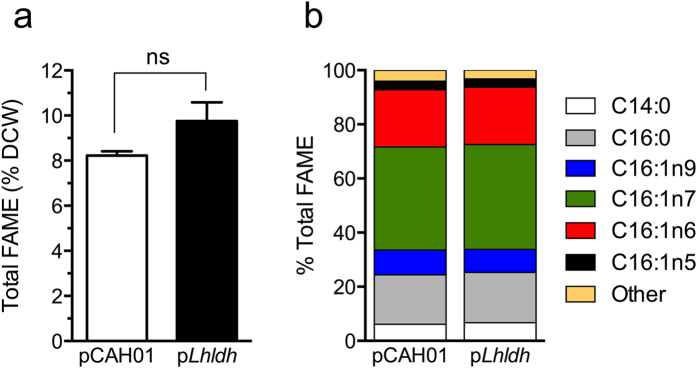
Fatty acid methyl ester (FAME) compositional analysis (**a**) % FAME based on dry cell weight, and (**b**) representative fatty acid composition (%, w/w) of *M. buryatense* harboring an empty vector (pCAH01), or ectopically expressing heterologous, *Lactobacillus helveticus* LDH (p*LhLDH*) after 96 h of growth in a 5.0 L bioreactor.

Methanotrophs are being considered for production of biomass-derived fuels from CH_4_ due to their ability to accumulate intracellular lipids^2^,^3^,^4^. Based on this interest, we evaluated the effect of lactate production on cellular lipid concentration. Fatty acid methyl ester (FAME) analysis indicated that carbon was not diverted from potential methanotroph-derived lipid fuel precursors while concurrently producing lactate, as both the *pLhldh* and control strains displayed similar cellular lipid content and profiles, composed primarily of hexadecanoic and hexadecenoic acids (Fig. 6a,b). Collectively, these data support the potential for concurrent methane biocatalysis to a platform chemical and fuel precursors from residual biomass.

## Discussion

CH_4_ from natural gas is currently flared or vented globally, resulting in large greenhouse gas emissions^2^ and revenue and energy losses^2^,^7^. Additionally, CH_4_-rich biogas derived from energy crops, crop residues, biofuel residues (such as stillage and glycerol), manure, and other organic waste streams, through anaerobic digestion in agricultural/food waste digesters, waste water treatment plants (WWTP), and landfills, offers a versatile, high-volume, renewable source of CH_4_^28^,^29^.

Biological production of value-added chemicals from CH_4_ represents a path to concurrently mitigate greenhouse gas emissions and utilize an abundant-yet-underutilized feedstock. Halotolerant alkaliphilic methanotrophs are promising biocatalysts for this purpose, displaying relatively rapid growth rates, high culture densities, recalcitrance to toxic components in biogas and natural gas, and possessing well-characterized central metabolic pathways^18^,^19^,^30^. Based on these characteristics, we employed *M. buryatense* for the bioconversion of CH_4_ to the platform chemical, lactate. We constructed an inducible broad host-range gene expression vector containing the tetracycline promoter/operator, which can be used in an array of methanotrophs that replicate the *oriV* origin of replication. Using these tools, we demonstrated microbial conversion of CH_4_ to lactate, a high-volume biochemical precursor predominantly utilized for the production of bioplastics, by an engineered methanotroph expressing a heterologous *Lactobacillus* LDH. The data presented herein provide proof-of-concept for bioconversion of CH_4_ to an industrial platform chemical using an engineered methanotrophic bacterium, but also highlight some of the hurdles associated with CH_4_ biocatalysis, such as CH_4_ assimilation rate (discussed further below).

The maximum lactate titer produced by the p*Lhldh M. buryatense* reached 1.3 g lactate/L. Currently, lactate produced from pure sugars or lignocellulosic hydrolysates utilizing metabolically-engineered industrial microbes reach titers approximately 100-fold greater than those achieved here^31^,^32^, leaving significant room for further *M. buryatense* metabolic engineering. Notably, however, this titer is over one order of magnitude higher than those achieved for any previously reported engineered methanotrophic bioproduct^13^, and comparable to titers achieved for an array of fuels and chemicals from biomass-derived substrates in proof-of-principle investigations^33^,^34^,^35^.

Our data show that CH_4_ uptake by the organism limits productivity in a continuous stirred tank bioreactor. Indeed, the engineered p*Lhldh* strain only assimilated ~2% (g/g) of the supplied CH_4_ in a 5 L bioreactor (Fig. 5), potentially due to limited gas-to-liquid transfer and/or methanotrophic methane oxidation. Moreover, *M. buryatense* converts 50–60% (g/g) of the assimilated CH_4_ to biomass (Figure S2). Considering that >10% (g/g) carbon is converted to other excreted products, including acetate, formate, and CO_2_ (Figure S2), 60% (g/g) of carbon is converted to biomass (Figure S2), and it is estimated that up to 25% of carbon is utilized to synthesize exopolysaccharide^36^, ~5% (g/g) of carbon (~0.6 g CH_4_/24 h based on Fig. 5B) is available for conversion to lactate in a process coupled to cell growth. Based on these metrics (Figure S3), >75% of the available carbon was converted to the target product under our experimental conditions. Given the large proportion of carbon flux diverted to non-target products, bioprocess designs that decouple biomass and bioproduct accumulation (such as 2-stage fermentation or cell immobilization), novel bioreactors, and hypothesis-driven strain-engineering strategies will be required to optimize CH_4_ assimilation and flux to product. Ongoing studies are focused on increasing the tolerance of *M. buryatense* to lactate, and rational strain engineering to increase CH_4_ biocatalysis by enhancing rates of CH_4_ consumption, carbon conversion efficiency, and carbon flux to pyruvate. Technoeconomic analysis is also currently underway to define the minimum productivity metrics required for a gas-to-liquid bioprocess to be competitive with conventional sugar-based lactate production.

Additional potential and hurdles associated with bioconversion of methane to liquid fuels has recently been extensively reviewed^2^,^3^,^4^,^7^. Several methanotrophic bacteria possess relatively high lipid content due to the accumulation of intracytoplasmic membrane to accommodate the particulate methane monooxygenase^37^. This intrinsically high lipid content makes these organisms attractive platforms for production of fatty-acid derived fuels^2^. Indeed, the relatively high biomass yield and lipid content of *M. buryatense* presented here support that these organisms have potential for lipid-derived fuels. Importantly, lactate production did not alter methanotroph lipid content or speciation. This suggests that the *pLhldh* strain maintains flux to lipids while also producing lactate, either by increasing flux to pyruvate or diverting additional acetyl-CoA to lipid biosynthesis. However, it is worth noting that we did observe a modest decrease in p*Lhldh* growth compared to the control strain (Fig. 4), presumably due to elevated LDH activity. Thus, an increase in lactate titer may have a more pronounced effect on cell growth since LDH could deplete the pyruvate pool. Nonetheless, data presented here indicate future studies targeting co-production of fuels and chemicals using methanotrophic bacteria are warranted.

CH_4_ biocatalysis offers a means to concurrently liquefy and upgrade CH_4_, enabling its utilization in conventional transportation and industrial manufacturing infrastructure. This work also raises the possibility of syngas-derived CH_3_OH valorization^38^, expanding the renewable substrates available for methanotrophic biocatalysis. Producing chemicals and fuels from CH_4_ expands the suite of products generated from biorefineries, municipalities, and agricultural operations, with the potential to increase revenue and reduce greenhouse gas emissions. By integrating this process into a conventional biorefinery, new opportunities for recycling and other cost reductions will become apparent.

## Materials and Methods

### Plasmid construction and transformation

Strains and plasmids used in this study are presented in Table S2. Plasmids for heterologous gene expression were constructed using 2X Gibson Assembly Mix from New England Biolabs (Ipswich, MA) following the manufacturers protocol. Polymerase chain reactions were performed using Q5 High-Fidelity Polymerase from New England Biolabs and primers (Table S3) purchased from Integrated DNA Technologies (Coralville, IO). The inducible, broad-host range vector pCAH01 was constructed by fusing the tetracycline promoter/operator (tet^p/o^) from pASK75 with the IncP-containing pAWP78 backbone^22^,^23^. Codon-optimized LDH genes from *B. longum*, *L. helveticus*, and *E.coli* (Table S1) were synthesized by Integrated DNA Technologies. Synthetic *ldh* genes and the native *M. buryatense ldh* (METBUDRAFT_3726) were amplified with a 3′ Flag-tag via PCR and cloned directly downstream of the tet^p/o^, generating plasmids pCAH01::Mb*ldhFlag*, pCAH01::Lh*ldhFlag*, pCAH01::Bl*ldhFlag*, pCAH01::Ec*ldhFlag*. Final constructs were confirmed by sequence analysis (Genewiz, South Plainfield, NJ). *Escherichia coli* Zymo 5a (Zymo Research, Irvine, CA) was used for cloning and plasmid propagation, and *E. coli* S17-1 λpir was used as the conjugation donor strain. *E.coli* strains were grown at 37 °C in Luria-Bertani (LB) broth supplemented with 50ug/mL of kanamycin. Plasmid constructs were transformed into *M. buryatense* via conjugation as previously described^22^. Positive transformants selected on NMS2 agar containing 50 μg/mL of kanamycin were confirmed using plasmid-specific primers in polymerase chain reactions.

### Cultivation and growth parameters

*M. buryatense* 5GB1S were routinely cultured in NMS2 medium at 30 °C with orbital shaking at 175 rpm as previously described^22^,^39^. Strains were grown in sealed 1 L glass serum bottles (Kimble Chase, Vineland, NJ) with 20% (v/v) CH_4_ in air, or 500 mL baffled flasks supplemented with 1% CH_3_OH (v/v). Serum bottle and shake flask cultures were inoculated at OD_600_ = 0.01 with plate-harvested biomass. LDH expression was induced by adding 0.5–2.0 μg/mL anhydrotetracycline (aTc, Sigma-Aldrich, St. Louis, MO) at the time of inoculation. To determine the minimum inhibitory concentration, 1% CH_3_OH (v/v) NMS2 was supplemented with increasing concentrations of aTc or sodium lactate (Sigma-Aldrich) and growth was monitored by measuring the OD_600_ using a spectrophotometer.

### GFP fluorescence

A synthetic emerald GFP (Life Technologies, Carlsbad, CA) open reading frame was amplified by PCR and cloned into pCAH01 via Gibson assembly to generate pCAH01::*emGFP. M. buryatense* harboring the pCAH01::*emGFP* vector was subcultured (OD_600_ = 0.01) in 1% CH_3_OH (v/v) NMS2 and induced with increasing concentrations of aTc. After 24 h of induction, 100 μL of culture samples were aliquoted into a Nunc™ F96 MicroWell™ Plate (Thermo Scientific, Waltham, MA) and fluorescence was measured (485 nm_ex_/520 nm_em_) using a FLUOstar Omega fluorometer (BMG LABTECH, Cary, NC).

### Western blotting

5GB1S harboring pCAH01::Mb*ldhFlag*, pCAH01::Lh*ldhFlag*, pCAH01::Bl*ldhFlag*, pCAH01::Ec*ldhFlag*, or the empty pCAH01 vector were grown in NMS2 supplemented with 1% CH_3_OH (v/v) and induced with 0.5 μg/mL aTc. Samples were taken at 24 h post-induction, pelleted, resuspended in lysis buffer, and disrupted by sonication. Samples normalized to 500 ng total protein were resolved using 12% (v/v) SDS-PAGE, transferred electrophoretically to a PVDF membrane, and immunoblotted with the anti-FLAG M2 monoclonal antibody (Sigma-Aldrich).

### Enzyme Assays

Cell free extracts for LDH activity measurements were prepared by sonication of logarithmically growing *M. buryatense* or *L. helveticus* cultures with or without LDH induction. Protein concentrations were determined with the Pierce 660nm Protein Assay Reagent (Life Technologies) using bovine serum albumin as a protein standard. Reactions were initiated by the addition of 50–500 μg lysate to reaction buffer consisting of 0.2 M Tris HCl, pH 7.5, 10 mM NADH, and 30 mM sodium pyruvate at 30 °C. LDH activities are expressed in units (U) per milligram of protein where one U was defined as the amount of enzyme required to oxidize 1 *μ*mol of NADH per min measured at A_340nm_.

### Bioreactor fermentations

CH_4_ fermentations were performed in a 0.5 L Biostat-Q Plus (Sartorius Stedim Biotech) or 5.0 L BioFlo batch bioreactor (New Brunswick Scientific, Edison, NJ) containing NMS2 medium supplemented with 8X KNO_3_, 2X phosphate buffer, and 4X trace element solution to support high cell growth. The bioreactor was inoculated at OD_600_ = 2 with a CH_4_-grown seed culture grown in NMS2 containing 2X KNO_3_. 2 μg/mL aTc was immediately added to induce LDH expression. The temperature was maintained at 30 °C, and mixing was achieved by using a bottom marine impeller and mid-height Rushton impeller at 500 rpm. A continuous flow rate of 300 ccm (0.5 L) or 300αcm (5.0 L) 20% (v/v) methane in air was maintained. Antifoam was added as needed. CH_4_ off-gas was measured every 20 min for the duration of bioreactor fermentations by using an infrared-based BlueSens methane detector (Herten, Germany). CH_4_ consumption was determined by calculating the difference between off-gas detected before and after inoculation. % CH_4_ consumption was converted to weight based on CH_4_ density and the flow rate (56.64 g CH_4_/24 h, 0.5 L reactor; 566.4 g CH_4_/24 h, 5.0 L reactor).

### Compositional analysis of culture supernatants

High pressure liquid chromatography (HPLC) was used to detect lactate, formate, acetate, and CH_3_OH in culture supernatants. At the indicated time, the OD_600_ was measured and a 1 mL sample was taken for HPLC analysis. Culture supernatant was filtered using a 0.2 μm syringe filter or 0.5 mL 10 K MWCO centrifuge tube (Life Technologies) and then separated using a model 1260 HPLC (Agilent, Santa Clara, CA) and a cation H HPx-87H column (Bio-Rad). A 0.1 mL injection volume was used in 0.01 N sulfuric acid with a 0.6 mL/min flow rate at 55 °C. DAD detection was measured at 220 nm and referenced at 360 nm, and organic acid concentrations were calculated by regression analysis compared to known standards.

### FAME analysis

Whole biomass lipid content was measured as fatty acid methyl esters (FAMEs), as described previously^40^. Briefly, 7 to 10 mg of lyophyilized biomass (dried overnight at 40 °C under vacuum) was homogenized with 0.2 mL of chloroform:CH_3_OH (2:1, v/v), and the resulting solubilized lipids were transesterified *in situ* with 0.3 mL of HCl:CH_3_OH (5%, v/v) for 1 h at 85 °C in the presence of a known amount of tridecanoic acid (C13) methyl ester as an internal standard. FAMEs were extracted with hexane (1 mL) at room temperature for 1 h and analyzed by gas chromatography:flame ionization detection (GC:FID) on a DB-WAX column (30 m × 0.25 mm i.d. and 0.25 μm film thickness).

### Statistical analysis

Data between two groups were analyzed using an unpaired, two-tailed *t*-test. Determination of statistical significance between multiple comparisons was achieved using one-way analysis of variance (ANOVA) followed by a Dunnett’s post-test. Data were considered statistically significant when *p* < 0.05.

## Additional Information

**How to cite this article**: Henard, C. A. *et al.* Bioconversion of methane to lactate by an obligate methanotrophic bacterium. *Sci. Rep.* **6**, 21585; doi: 10.1038/srep21585 (2016).

## Supplementary Material

Supplementary Information

## Figures and Tables

**Table 1:** Lactate dehydrogenase activity.

Strain	μmol NADH/min (U)	U/mg
5GB1S	0.006 ± 0.0005	0.060 ± 0.005
5GB1S *pLhldh* uninduced	0.009 ± 0.0005	0.087 ± 0.005
5GB1S *pLhldh* aTc*	0.044 ± 0.006	0.435 ± 0.062
*L. helveticus*	0.025 ± 0.009	2.454 ± 0.891

^*^LDH expression was induced with 0.5 μg/mL anhydrotetracycline (aTc) for 24 h. The data represent the mean ± SD of 3 independent observations.

**Table 2:** Lactate titer, yield, and productivity in stirred bioreactors.

CSTR Culture size	CH_4_ consumed (g, 96 h)	Titer (g/L, 96 h)	Yield (g/g)	Productivity (g/L/h)
0.3 L*	4.06	0.12	0.009	0.0013
3.0 L*	49.0	0.81	0.050	0.0084

^*^data based on the mean lactate titer presented in Fig. 4 and CH_4_ consumption data presented in Fig. 5.
