# Supplementary material for: Bioconversion of methane to lactate by an obligate methanotrophic bacterium
Source: Sci Rep. 2016 Feb 23;6:21585. doi: 10.1038/srep21585 (PMC4763203; doi:10.1038/srep21585)
Supplement: Supplementary Information [file srep21585-s1.pdf]

Bioconversion of methane to lactate by an obligate methanotrophic bacterium.

Calvin A. Henard[1], Holly Smith[1], Nancy Dowe[1], Marina G. Kalyuzhnaya[2], Philip T. Pienkos[1], and Michael T. Guarnieri[1*]

[1]National Bioenergy Center, National Renewable Energy Laboratory, Golden, CO USA 80401

[2]Department of Biology, San Diego State University, San Diego, CA USA 92182

Supplementary Material

Supplemental Material: Henard, et al. Bioconversion of methane to lactate by an obligate methanotrophic bacterium.

Table S1. Codon-optimized lactic acid dehydrogenase genes.

	**Optimized sequence**
Lactobacillus helveticus ldh	ATGGCGCGTGAAGAGAAACCGCGTAAAGTCATCCTGGTCGGCGA<br>CGGCGCTGTCGGCTCGACGTTCGCGTTCTCGATGGTCCAACAAG<br>GTATCGCGGAAGAACTGGGCATCATCGACATCGCGAAAGAACAT<br>GTCGAAGGCGACGCGATCGACCTGGCGGACGCGACGCCGTGGA<br>CGTCGCCG*AAAAAC*ATCTACGCGGCGGACTACCCGGACTGCAAA<br>GACGCGGACCTGGTCGTCATCACGGCGGGCGCGCCGCAAAAAC<br>CGGGCGAAACGCGTCTGGACCTGGTCAACAAAAACCTGAAAATC<br>CTGTCGTCGATCGTCGAACCGGTCGTCGAATCGGGCTTCGAAGG<br>CATCTTCCTGGTCGTCGCGAACCCGGTCGACATCCTGACGCATG<br>CGACGTGGCGTATGTCGGGCTTCCCGAAAGACCGTGTCATCGGC<br>TCGGGCACGTCGCTGGACACGGGCCGTCTG*CAAAAA*GTCATCGG<br>CAAAATGG*AAAA*CGTCGACCCGTCGTCGGTCAACGCGTACATGC<br>TGGGCGAACATGGCGACACGGAATTCCCGGCGTGGTCGTACAAC<br>AACGTTGCGGGCGTCAAAGTCGCGGACTGGGTCAAAGCGCATAA<br>CATGCCGGAATCGAAACTGGAAGACATCCATCAAGAAGTCAAAGA<br>CATGGCGTACGACATCATCAACAAAAAAGGCGCGACGTTCTACG<br>GTATCGGTACGGCGTCGGCGATGATCGCGAAAGCGATCCTGAAC<br>GACGAACATCGTGTCCTGCCGCTGTCGGTTCCGATGGACGGCGA<br>ATACGGCCTGCATGACCTGCATATCGGCACGCCGGCGGTCGTTG<br>GCCGTAAAGGCCTGGAACAAGTTATCGAAATGCCGCTGTCGGAC<br>AAAGAACAAGAACTGATGACGGCGTCGGCGGACCAACTGAAAAA<br>AGTTATGGACAAAGCGTTCAAAGAAACGGGCGTTAAAGTCCGTCA<br>ATAA
Bifidobacterium longum ldh	ATGGCGGAAACGACGGTTAAACCGACGAAACTGGCGGTTATCGG<br>CGCGGGCGCGGTTGGCTCGACGCTGGCGTTCGCGGCGGCGCAA<br>CGTGGCATCGCGCGTGAAATCGTTCTGGAAGACATCGCGAAAGA<br>ACGTGTTGAAGCGGAAGTTCTGGACATGCAACATGGCTCGTCGTT<br>CTACCCGACGGTTTCGATCGACGGCTCGGACGACCCGGAAATCT<br>GCCGTGACGCGGACATGGTTGTCATCACGGCGGGCCCGCGTCA<br>AAAACCGGGCCAATCGCGTCTGGAACTGGTTGGCGCGACGGTTA<br>ACATCCTGAAAGCGATCATGCCGAACCTGGTTAAAGTTGCGCCGA<br>ACGCGATCTACATGCTGATCACGAACCCGGTTGACATCGCGACG<br>CATGTTGCGCAAAAACTGACGGGCCTGCCGGAAAACCAAATCTTC<br>GGTTCGGGCACGAACCTGGACTCGGCGCGTCTGCGTTTCCTGAT<br>CGCGCAACAAACGGGCGTTAACGTTAAAAACGTTCATGCGTACAT<br>CGCGGGCGAACATGGCGACTCGGAAGTTCCGCTGTGGGAATCG<br>GCGACGATCGGCGGCGTTCCGATGTGCGACTGGACGCCGCTGC<br>CGGGCCATGACCCGCTGGACGCGGACAAACGTGAAGAAATCCAT<br>CAAGAAGTTAAAAACGCGGCGTACAAAATCATCAACGGCAAAGGC<br>GCGACGAACTACGCGATCGGCATGTCGGGCGTTGACATCATCGA<br>AGCGGTTCTGCATGACACGAACCGTATCCTGCCTGTTTCTTCGAT<br>GCTGAAAGACTTCCATGGTATCTCTGACATCTGTATGTCTGTTCCT

	ACTCTGCTGAATCGTCAAGGTGTTAACAACACGATCAACACGCCT<br>GTTTCTGACAAAGAACTGGCTGCTCTGAAACGTTCGGCTGAAACT<br>CTGAAAGAAACTGCTGCTCAATTCGGTTTCTGA
Escherichia coli ldhA	ATGAAACTGGCGGTCTACTCGACGAAACAATACGACAAAAAATAC<br>CTGCAACAAGTCAACGAATCGTTCGGCTTCGAACTGGAATTCTTC<br>GCTTCCTGCTGACGGAAAAAACGGCGAAAACGGCGAACGGCTGC<br>GAAGCGGTCTGCATCTTCGTCAACGACGACGGCTCGCGTCCGGT<br>CCTGGAAGAACTGAAAAAACATGGCGTCAAATACATCGCGCTGC<br>GTTGCGCGGGCTTCAACAACGTCGACCTGGACGCGGCGAAAGAA<br>CTGGGCCTGAAAGTCGTCCGTGTCCCGGCGTACGACCCGGAAG<br>CGGTCGCGGAACATGCGATCGGCATGATGATGACGCTGAACCGT<br>CGTATCCATCGTGCGTACCAACGTACGCGTGACGCGAACTTCTC<br>GCTGGAAGGCTGACGGGCTTCACGATGTACGGCAAAACGGCGG<br>GCGTCATCGGCACGGGCAAAATCGGCGTCGCGATGCTGCGTATC<br>CTGAAAGGCTTCGGCATGCGTCTGCTGGCGTTCGACCCGTACCC<br>GTCGGCGGCGGCGCTGGAACTGGGCGTCGAATACGTCGACCTG<br>CCGACGCTGTTCTCGGAATCGGACGTCATCTCGCTGCATTGCCC<br>GCTGACGCCGGAAAACTACCATCTGCTGAACGAAGCGGCGTTCG<br>AACAAATGAAAAACGGCGTCATGATCGTCAACACGTCGCGTGGC<br>GCGCTGATCGACTCGCAAGCGGCGATCGAAGCGCTGAAAAACCA<br>AAAAATCGGCTCGCTGGGCATGGACGTCTACGAAAACGAACGTG<br>ACCTGTTCTTCGAAGACAAATCGAACGACGTCATCCAAGACGACG<br>TCTTCCGTCGTCTGTCGGCGTGCCATAACGTCCTGTTCACGGGC<br>CATCAAGCGTTCCTGACGGCGGAAGCGCTGACGTCGATCTCGCA<br>AACGACGCTGCAAAACCTGTCGAACCTGGAAAAAGGCGAAACGT<br>GCCCGAACGAACTGGTCTAA

Table S2. Strains and Plasmids

Strains	Genotype/Description	References
Methylomicrobium buryatense 5GB1S	Wild-type	(1)
E. coli Zymo 5α	F^{-}φ80lacZΔM15 Δ(lacZYA-argF)U169 deoR nupG recA1 endA1 hsdR17(r_K^- m_K^+) phoA glnV44 (supE44) thi-1 gyrA96 relA1, λ-	Zymo Research
E. coli S17-1 λpir	Tp^r Sm^r *recA thi pro hsd*(r^- m^+)RP4-2-Tc::Mu::Km Tn*7* λ*pir*	ATCC
**Plasmids**		
pASK75	P_{tetA} *bla-tetR* CoE1*ori* F1	(2)
pAWP78	*oriV oriT trfA ahp*	(1) Addgene # 61263
pCAH01	P_{tetA} *bla-tetR* CoE1*ori* F1 *oriV oriT trfA ahp*	This study
pCAH01::*emGFP*	*emGFP* inserted downstream of P_{tet} using Gibson assembly	This study
pCAH01::*MbldhFlag*	*M. buryatense* lactate dehydrogenase inserted downstream of P_{tet} using Gibson assembly	This study
pCAH01::*EcldhAFlag*	Synthetic *Escherichia coli* lactate dehydrogenase inserted downstream of P_{tet} using Gibson assembly	This study
pCAH01::*LhldhFlag* *(pLhldh)*	Synthetic *Lactobacillus helveticus* lactate dehydrogenase inserted downstream of P_{tet} using Gibson assembly	This study
pCAH01::*BlldhFlag*	Synthetic *Bifidobacterium longum* lactate dehydrogenase inserted downstream of P_{tet} using Gibson assembly	This study

Table S3. Primers

Name	Primers (5' to 3')	Usage
oCAH146<br>oCAH147	F:cagaccccgtagaaaaTTGTCGGGAAGATGCGTG<br>R:gaaccgtaaaaaggccCAGCTCACTCAAAGGCGG	IncP origin from pAWP78
oCAH148<br>oCAH145	F:GGCCTTTTTACGGTTCCTG<br>R:TTTTCTACGGGGTCTGAC	pASK75 backbone with P_{tet} and *bla-tetR*
oCAH172<br>oCAH173	F:CCCGACACCATCGAATGGCCAGATG<br>R:CAGGGCGCGTGGAGATCCGT	pCAH01 sequencing
oCAH152<br>oCAH149	F:AAGCTTGACCTGTGAAGTG<br>R:TTCACTTTTCTCTATCACTGATAG	pCAH01 for Gibson Assembly
oCAH150<br>oCAH151	F:gtgatagagaaaagtgaaATGGTGAGCAAGGGCGAG<br>R:cttcacaggtcaagcttTTACTTGTACAGCTCGTCCATG	*emGFP*
oCAH168<br>oCAH169	F:gtgatagagaaaagtgaaATGAAAATAGCCATAATCGGAG<br>R:cttcacaggtcaagcttTCACTTGTCATCGTCATCCTTGTAATCTCTTTCACCTTTCACCTTTCCT	*Methylomicrobium buryatense ldh 1X FLAG*
oCAH170<br>oCAH171	F:gtgatagagaaaagtgaaATGAAACTGGCGGTCTACTC<br>R:cttcacaggtcaagcttTTACTTGTCATCGTCATCCTTGTAATCGACCAGTTCGTTCGGGCACG	Codon-optimized *Escherichia coli ldhA 1X FLAG*
oCAH142<br>oCAH165	F:gtgatagagaaaagtgaaATGGCGCGTGAAGAGAAAC<br>R:cttcacaggtcaagcttTTACTTGTCATCGTCATCCTTGTAATCTTGACGGACTTTAACGCC	Codon-optimized *Lactobacillus helveticus ldh 1X FLAG*
oCAH166<br>oCAH167	F:gtgatagagaaaagtgaaATGGCGGAAACGACGGTTAA<br>R:cttcacaggtcaagcttTTACTTGTCATCGTCATCCTTGTAATCGAAACCGAATTGAGCAGCAG	Codon-optimized *Bifidobacterium longum ldh 1X FLAG*

* Lowercase denotes homologous sequence for Gibson assembly

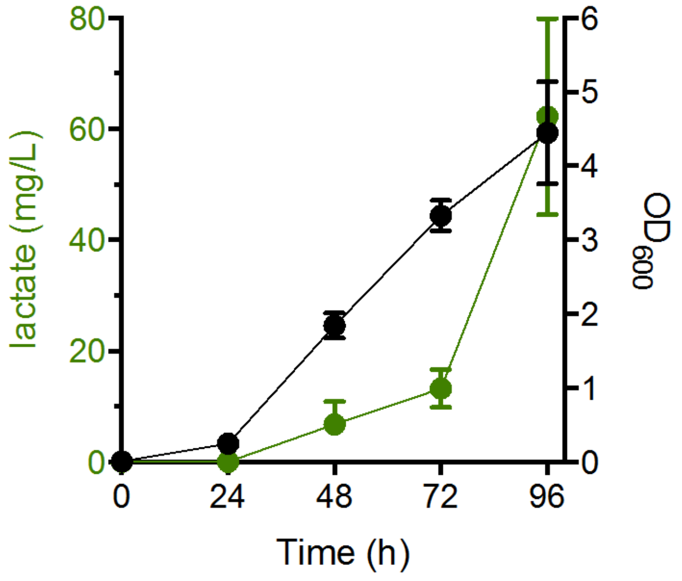

Figure S1. Bioconversion kinetics of methane to lactate. Growth (black) and lactate accumulation (green) in batch cultures of the *Lactobacillus helveticus* LDH-overexpressing methanotroph (p*Lhldh*) in stirred flasks with continuous CH_4 feed (20% v/v in air). The data represent the mean ± SEM from 3 independent experiments (n=4).

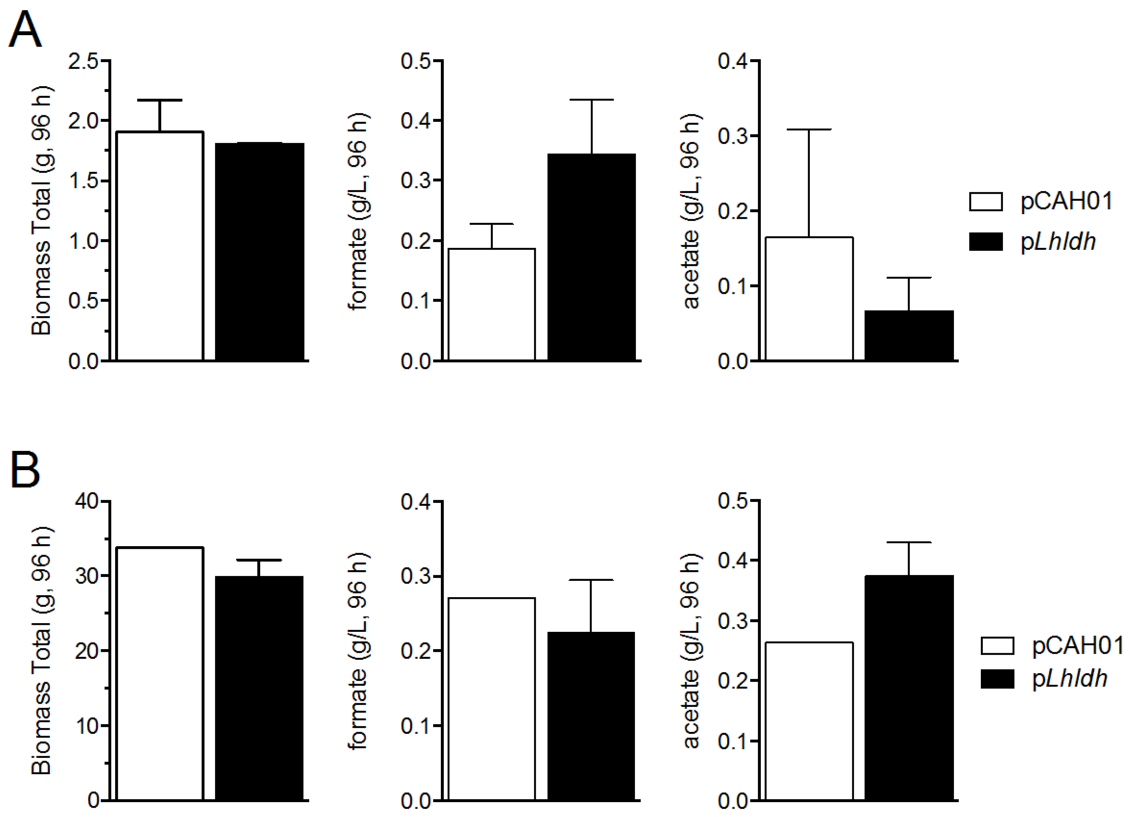

Figure S2. *M. buryatense* biomass and organic acid titers in a continuously stirred tank bioreactor.

Biomass, formate, and acetate production by engineered *M. buryatense* harboring an empty vector (pCAH01, white bars), or ectopically expressing heterologous *L. helveticus* LDH (p*LhLDH*, black bars) after 96 h of growth in 0.5 L methane bioreactor (A, 0.3 L culture volume) or 5.0 L methane bioreactor (B, 3.0 L culture volume). The p*Lhldh* data represent the mean ± SEM from at least two independent experiments (n=2-4), and pCAH01 data are from two independent 0.5 L bioreactor fermentations (n=2) and a single 5 L bioreactor experiment.

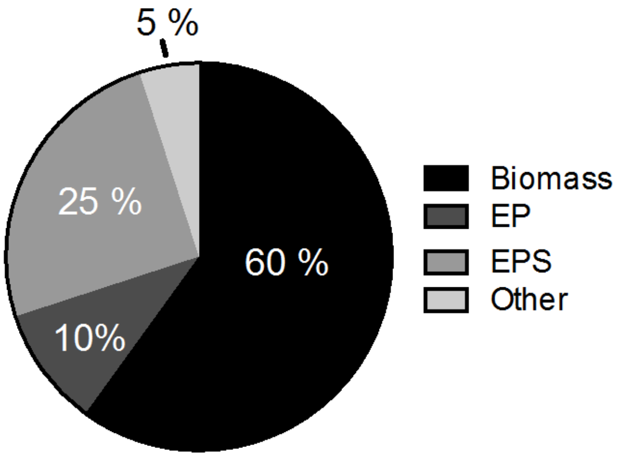

Figure S3. Carbon flux in an engineered lactate-producing *Methylomicrobium buryatense*. CH_4 amounts are based on the measured CH_4 consumed (49.0 g), dry-cell weight (29.8 g), and other excreted products (EP, including formate and acetate) during 96 h cultivation in a 5 L bioreactor. The % carbon utilized for exopolysaccharide (EPS) synthesis is based on published EPS yields (3).

1. **Puri AW**, **Owen S**, **Chu F**, **Chavkin T**, **Beck DAC**, **Kalyuzhnaya MG**, **Lidstrom ME**. 2015. Genetic Tools for the Industrially Promising Methanotroph *Methylomicrobium buryatense*. Appl Environ Microbiol **81**:1775–1781.

2. **Skerra A**. 1994. Use of the tetracycline promoter for the tightly regulated production of a murine antibody fragment in *Escherichia coli*. Gene **151**:131–135.

3. **Malashenko, Yu. R., Pirog, T. P., Romanovskaya, V. A., Sokolov, I. G. T. A. Grinberg.** 2001. Search for Methanotrophic Producers of Exopolysaccharides *Applied Biochemistry and Microbiology, Vol. 37, No. 6, 2001, pp. 599–602.*
